# Prevalence and incidence of peripheral neuropathy, peripheral artery disease, foot disease, and lower extremity amputation in people with diabetes in Ireland; a systematic review protocol.

**DOI:** 10.12688/hrbopenres.13823.2

**Published:** 2024-08-21

**Authors:** Sinead Kavanagh, Jennifer A. Pallin, Ann Sinead Doherty, Peter Lazzarini, Linda O'Keeffe, Claire M Buckley

**Affiliations:** 1 School of Public Health, University College Cork, Cork, County Cork, Ireland; 2 Department of Endocrinology, Bantry Hospital, Bantry, Cork, Ireland; 3 Department of General Practice, University College Cork, Cork, County Cork, Ireland; 4 School of Public Health and Social Work,, Queensland University of Technology, Brisbane, Queensland, Australia; 5 Allied Health Research Collaborative, The Prince Charles Hospital, Brisbane, Queensland, Australia

**Keywords:** Diabetic foot disease, foot ulceration, amputation, Irish health system, incidence, prevalence.

## Abstract

**Introduction:**

Internationally, the prevalence of diabetes is increasing, and with this comes an increase in diabetes related complications. Diabetic foot disease is the most common lower extremity complication in people with diabetes causing 2% of the global disease burden. It, is associated with major morbidity, mortality, and costs to health services. Despite this burden, the incidence and prevalence of diabetic foot disease is unknown in Ireland. This paper presents a protocol for a systematic review to examine the incidence and prevalence of diabetic foot disease in the Irish population.

**Methods:**

A systematic review will be performed using the Preferred Reporting Items for Systematic Review and Meta-Analyses guidelines. Pubmed, EMBASE, and Lenus, the Irish Health Research repository, will be searched for publications in any language and without restrictions to date. Title, abstract, and full text screening will be carried out independently by two investigators. Publications reporting on the incidence or prevalence of peripheral neuropathy, peripheral artery disease, ulceration, or amputation in people with diabetes in Ireland, from a defined geographical catchment area of Ireland, will be included. Joanna Briggs Institute (JBI) Critical Appraisal tool will be used to assess included studies methodological quality. Results will be reported in line with the Preferred Reporting Items for Systematic Review and Meta-Analyses guidelines.

**Conclusion:**

The results of this systematic review can be used to inform appropriate stakeholders on the incidence and prevalence of diabetic foot disease in Irish populations, enabling decision making around appropriate use of resources to help prevent, and improve management of this disease.

**Systematic review registration:**

CRD42023472904

## Introduction

Globally, the prevalence and incidence of type 1 and type 2 diabetes mellitus continues to increase ^1^. With an increase in diabetes prevalence, comes an increase in diabetes related macrovascular complications, including cardiovascular, cerebrovascular, and peripheral artery disease, and microvascular complications including retinopathy, peripheral neuropathy, and nephropathy. Peripheral neuropathy and peripheral artery disease are significant risk factors for diabetic foot ulcers and lower limb amputations, each of which place a significant negative burden on those affected and on health systems, resulting in increased risk of death, poorer quality of life and significant costs to health systems ^2– 8^. Diabetic foot disease has also found to cause 2% of the global disease burden ^9^. In addition, the risk of a person undergoing a lower-extremity amputation is 22 times higher in a person with diabetes when compared to a person without and up to 80% of diabetes related amputations are preceded by a diabetic foot ulcer ^10, 11^.

It is estimated that globally, up to 26.1 million people with diabetes will develop a diabetic foot ulcer ^12^. The risk of death in those with diabetic foot ulcers, is almost twice as high when compared to those with diabetes who have never experienced an ulcer ^2^. This risk of mortality is also higher in those who have higher levels of socio-economic deprivation ^13^. To reduce the morbidity and mortality associated with these complications, timely access to appropriate health services is necessary. In line with international recommendations, this involves identifying and referring those with risk factors for ulceration and amputation to appropriate diabetic foot prevention and management services ^14^. However, evidence suggests that many people are not being screened for risk factors and there is insufficient staff to deal with service demands on diabetic foot services ^15, 16^. One way of overcoming this is by ensuring enough resources (e.g., healthcare professionals, infrastructures, and finances) are in place to provide screening for risk factors, and implementation of prevention and management strategies for the foot at-risk of ulceration and amputation. To ensure this, up-to-date population level estimates of the incidence and prevalence of these complications are required to identify public health priorities and allow for appropriate health service planning. Previously published systematic reviews and meta-analyses have estimated a global prevalence of 6.3% for diabetic foot ulcers, with higher prevalence in North America (13%) and Africa (7.2%), and lower prevalence in Europe (5.1%), Asia (5.5%), and Oceania (3%) ^17^. Regarding diabetic foot disease, it is estimated that between 19–34% of people with diabetes will develop this throughout their lifetime, with further evidence suggesting that years lived with diabetic foot disease is increasing globally ^18^.

In Ireland a national diabetes registry does not exist. Thus, prevalence of diabetes and associated complications are based on estimates ^19^. Reports suggest that the incidence of diabetes related amputations is increasing ^20^, however, little is known about the incidence or prevalence of other diabetes related lower extremity complications. This study describes a protocol for a systematic review aiming to identify and synthesise the incidence and prevalence of diabetes related lower extremity complications in Irish populations.

## Methods

This protocol was developed using the Preferred Reporting Items for Systematic Reviews and Meta-Analysis Protocols Checklist (PRISMA-P), and the Joanna Briggs Institute Manual for systematic reviews ^21– 23^. The protocol has been registered prospectively on PROSPERO database for systematic reviews (registration number CRD42023472904).

### Study aim

To identify and synthesise the incidence and prevalence of diabetes related lower extremity complications in Irish populations.

### Objectives

To identify the following incidences and prevalence in Ireland:

1. Diabetes related peripheral neuropathy.
2. Diabetes related peripheral artery disease.
3. Diabetes related foot ulceration.
4. Diabetes related lower extremity amputations (total, major and minor).

### Eligibility criteria

- **Population:** Studies including people residing in the Republic of Ireland with a diagnosis of diabetes, and at least one of the following complications: peripheral neuropathy, peripheral artery disease, foot ulceration, and minor or major lower extremity amputation ^24^. Studies will be excluded if data relating to the Irish population cannot be separated from other population groups (e.g., those from the United Kingdom), and where we cannot not differentiate between those with and without a diagnosis of diabetes. The populations of interest for this review will need to be populations from a defined geographical catchment area of Ireland (e.g., national population, inpatient population etc) due to the requirement of a clearly defined denominator. Publication from a single centre(s) will be excluded, unless general or diabetes-population information for the selected centres is provided or if data is adjusted or compared to the overall Irish population demographics.
- **Settings:** Studies carried out in community settings (including GP settings, primary care centre etc) and hospital settings (including inpatient and outpatient) will be included.
- **Study design:** Prospective and retrospective cross-sectional, case-control or cohort studies will be included. Case reports, case series, and grey literature including reports published by public bodies and informal communications will be excluded. Also, studies that do not report original data will be excluded.

There will be no restrictions on year of publication and language.

### Outcomes

The outcomes of interest will be prevalence or incidence of peripheral neuropathy, peripheral artery disease, ulceration, and amputation, as defined by the International Working Group for the Diabetic Foot (IWGDF) ^24^.

### Search strategy

The following databases will be searched: ‘PubMed’, ‘EMBASE’, and the Irish Health Research repository ‘Lenus’ will be used. The Condition Context Population (CoCoPop) framework ^22^ was used to inform development of the search strategy, which was developed in Embase (See Box 1) and will be adapted for use in PubMed and the Irish Health Research repository ‘Lenus’. The search strategy will utilise keywords associated with the concepts of diabetic foot disease and incidence and prevalence. Boolean operators AND, OR and proximity operators will be utilised to combine search headings, where appropriate. Backward citation searching of articles selected for full text review will also be carried out.

**Box 1. Embase Search**

1. peripheral AND ('neuropathy'/exp OR neuropathy)
2. 'ischemia'/exp OR 'ischemia'
3. 'peripheral vascular disease'/exp OR 'peripheral vascular disease'
4. #2 OR #3
5. 'ulcer'/exp
6. 'foot'/exp
7. #5 AND #6
8. 'foot disease'/exp
9. 'diabetic foot'/exp
10. 'amputation'/exp
11. 'limb loss'/exp
12. #11 OR #11
13. #7 OR #8 OR #9
14. Incidence/exp
15. Prevalence/exp
16. #14 OR #15
17. 'ireland'/exp
18. 'irish (citizen)'/exp OR 'irish (citizen)'
19. #17 OR #18
20. 'diabetes mellitus'/exp

For peripheral neuropathy:

21. #1 AND #16 AND #20
22. #21 AND #19

For peripheral artery disease:

23. #4 AND #16 AND #20
24. #23 AND #19

For diabetic foot ulcers:

25. #13 AND #16 AND #19
26. #13 AND #19

For amputations:

27. #12 AND #19
28. #12 AND #16 AND #20
29. #27 AND #19

### Data management and study selection

***Software.*** Mendeley reference management system will be used as a bibliography manager, and Covidence ( https://www.covidence.org/) will be used for screening. The statistical Package Rev Man 5.4 will be used for meta-analysis.

***Study screening and selection.*** Results from each database will be imported into Mendeley, and at this stage any duplicates will be removed. Identified articles will then be exported to Covidence for title and abstract screening. This will be conducted independently by two reviewers. Any conflicts at this stage will be discussed, and where consensus cannot be reached, a third reviewer will be consulted. The same process will be employed for full text screening.

### Data extraction

Data extraction will be conducted by one reviewer using a standardised data extraction drafted form in Microsoft Excel then rechecked by a second reviewer for accuracy. Reviewers will not extract data from any studies in which they were an author or co-author to prevent any conflict of interest or potential bias. It will include the following details:

- Author and year
- Study design
- Study setting e.g., hospital or community setting.
- Year(s) of data collection
- Length of follow up
- Population characteristics including participant age, gender, diabetes type and diabetes duration, sociodemographics, living urban or rural and mobility levels
- Outcomes of interest
  ○ Peripheral neuropathy
  ○ Peripheral artery disease
  ○ Foot ulcer
  ○ Lower extremity amputation (total, minor or major)
- How outcomes were measured e.g., was it from medical chart review, clinical assessment, self-reported.

### Quality assessment

The most appropriate Joanna Briggs Institute (JBI) Critical Appraisal tool will be used to assess included studies methodological quality and establish the degree to which bias was addressed in the study’s design, conduct, and analysis ^22, 25^. To assess these parameters, the JBI checklists offer a series of questions to which ‘Yes’, ‘No’, ‘Unclear’, and ‘Not applicable’ are the provided answers. Assessment will be carried out by two reviewers independently, and where disagreements occur, a third reviewer will be consulted.

### Presenting and reporting of results

A PRISMA flow diagram will be included to illustrate the study selection process. Tables will be used to allow for presentation of study characteristics, quality assessment scores and reporting of incidence and prevalence. In addition, data will be synthesised using a narrative synthesis approach to allow for comparison of similarities and the disparities between the findings of identified studies. This includes comparisons of results (prevalence and incidence of outcome of interest) between study settings, participant characteristics, measurement of outcomes, and methodological quality of included studies. In addition, if possible, subgroup comparison between national and regional studies will also be conducted. A meta-analysis will be performed if at least three publications reporting on the same outcome of interest, using similar definitions to identify the outcome, in a similar population of interest, with data collected within 10 years of each other are found. Meta-analyses will be used to calculate pooled incidence or prevalence estimates using a random-effects model, with the I ^2^ test used to examine heterogeneity across publications.

## Conclusion

The systematic review outlined in this protocol will synthesise the existing evidence on incidence and prevalence of peripheral neuropathy, peripheral artery disease, ulceration, and amputation in people with diabetes residing in the Republic of Ireland. It is believed this review will help inform stakeholders on the burden of diabetic foot disease and help inform them on the appropriate use of resources to reduce the burden associated with diabetic foot disease in Ireland.

## Study status

The protocol was registered prospectively with Prospero (CRD42023472904).

## Ethics

Ethical approval is not required for a systematic review.

## Data Availability

No data are associated with this article. OSF: PRISMA-P checklist for ‘Prevalence and incidence of peripheral neuropathy, peripheral artery disease, foot disease and lower extremity amputation in people with diabetes in Ireland; a systematic review protocol’. See: https://doi.org/10.17605/OSF.IO/THD78

## References

[1] Sun H Saeedi P Karuranga S : IDF Diabetes Atlas: global, regional and country-level diabetes prevalence estimates for 2021 and projections for 2045. *Diabetes Res Clin Pract.* 2022;183: 109119. 10.1016/j.diabres.2021.109119 34879977 PMC11057359

[2] Saluja S Anderson SG Hambleton I : Foot ulceration and its association with mortality in diabetes mellitus: a meta-analysis. *Diabet Med.* 2020;37(2):211–218. 10.1111/dme.14151 31613404

[3] Ribu L Birkeland K Hanestad BR : A longitudinal study of patients with diabetes and foot ulcers and their Health-Related Quality of Life: wound healing and quality-of-life changes. *J Diabetes Complications.* 2008;22(6):400–407. 10.1016/j.jdiacomp.2007.06.006 18413188

[4] Sekhar MS Thomas RR Unnikrishnan MK : Impact of diabetic foot ulcer on Health-Related Quality of Life: a cross-sectional study. *Semin Vasc Surg.* 2015;28(3–4):165–171. 10.1053/j.semvascsurg.2015.12.001 27113283

[5] Brownrigg JRW Davey J Holt PJ : The association of ulceration of the foot with cardiovascular and all-Cause mortality in patients with diabetes: A meta-analysis. *Diabetologia.* 2012;55(11):2906–2912. 10.1007/s00125-012-2673-3 22890823

[6] Kerr M Barron E Chadwick P : The cost of diabetic foot ulcers and amputations to the National Health Service in England. *Diabet Med.* 2019;36(8):995–1002. 10.1111/dme.13973 31004370

[7] Gillespie P Kelly L Hurley L : The effect of foot ulcers on costs of care for people with diabetes in Ireland. *The Diabetic Foot Journal.* 2014;17(3):107–12. Reference Source

[8] Gooday C Hardeman W Game F : A qualitative study to understand people’s experiences of living with Charcot neuroarthropathy. *Diabet Med.* 2022;39(6): e14784. 10.1111/dme.14784 34985149 PMC9305882

[9] Lazzarini PA Raspovic KM Meloni M : A new declaration for feet’s sake: Halving the global diabetic foot disease burden from 2% to 1% with next generation care. *Diabetes Metab Res Rev.* 2024;40(3): e3747. 10.1002/dmrr.3747 37997627

[10] Pecoraro RE Reiber GE Burgess EM : Pathways to diabetic limb amputation. basis for prevention. *Diabetes Care.* 1990;13(5):513–521. 10.2337/diacare.13.5.513 2351029

[11] Buckley CM O’Farrell A Canavan RJ : Trends in the incidence of lower extremity amputations in people with and without diabetes over a five-year period in the republic of Ireland. *PLoS One.* 2012;7(7): e41492. 10.1371/journal.pone.0041492 22859991 PMC3409236

[12] Edmonds M Manu C Vas P : The current burden of diabetic foot disease. *J Clin Orthop Trauma.* 2021;17:88–93. 10.1016/j.jcot.2021.01.017 33680841 PMC7919962

[13] Anderson SG Shoo H Saluja S : Social deprivation modifies the association between incident foot ulceration and mortality in type 1 and type 2 diabetes: a longitudinal study of a primary-care cohort. *Diabetologia.* 2018;61(4):959–967. 10.1007/s00125-017-4522-x 29264632 PMC6448990

[14] Bus SA Sacco ICN Monteiro Soares M : Guidelines on the prevention of foot ulcers in persons with diabetes IWGDF 2023 update. *Diabetes Metab Res Rev.* 2024;40(3): e3651. 10.1002/dmrr.3651 37302121

[15] Pankhurst CJW Edmonds ME : Barriers to foot care in patients with diabetes as identified by healthcare professionals. *Diabet Med.* 2018;35(8):1072–1077. 10.1111/dme.13653 29696678

[16] O’Neill K Riordan F Racine E : Adoption and initial lmplementation of a national integrated care programme for diabetes: a realist evaluation. *Int J Integr Care.* 2022;22(3): 3. 10.5334/ijic.5815 35891626 PMC9284993

[17] Zhang P Lu J Jing Y : Global epidemiology of diabetic foot ulceration: a systematic review and meta-analysis †. Ann Med. 2017;49(2):106–116. 10.1080/07853890.2016.1231932 27585063

[18] Zhang Y Lazzarini PA McPhail SM : Global disability burdens of diabetes-related lower-extremity complications in 1990 and 2016. *Diabetes Care.* 2020;43(5):964–974. 10.2337/dc19-1614 32139380

[19] Diabetes Ireland: Diabetes Prevalence in Ireland.Published 2022. Accessed July 31, 2023.

[20] Diabetes Ireland: Diabetes Footcare Statistics 2017-2021. 2022; Accessed July 31, 2023.

[21] Page MJ McKenzie JE Bossuyt PM : The PRISMA 2020 statement: an updated guideline for reporting systematic reviews. *BMJ.* 2021;372: n71. 10.1136/bmj.n71 33782057 PMC8005924

[22] Aromataris E Munn Z : JBI Manual for Evidence Synthesis. Joanna Briggs Institute. 10.46658/JBIMES-20-01

[23] Shamseer L Moher D Clarke M : Preferred reporting items for systematic review and meta-analysis protocols (PRISMA-P) 2015: elaboration and explanation. *BMJ.* 2015;349: g7647. 10.1136/bmj.g7647 25555855

[24] Van Netten JJ Bus SA Apelqvist J : Definitions and criteria for diabetes-related foot disease (IWGDF 2023 update). *Diabetes Metab Res Rev.* 2024;40(3): e3654. 10.1002/dmrr.3654 37186781

[25] Barker TH Stone JC Sears K : Revising the JBI quantitative critical appraisal tools to improve their applicability: an overview of methods and the development process. *JBI Evid Synth.* 2023;21(3):478–493. 10.11124/JBIES-22-00125 36121230

