# Supplementary material for: Prevalence and incidence of peripheral neuropathy, peripheral artery disease, foot disease, and lower extremity amputation in people with diabetes in Ireland; a systematic review protocol
Source: HRB Open Res. 2024 Aug 21;7:1. Originally published 2024 Jan 3. [Version 2] doi: 10.12688/hrbopenres.13823.2 (PMC11561379; doi:10.12688/hrbopenres.13823.2)
Supplement: Peer review history

# Reviewer report: Reviewer response for version 2

**DOI:** 10.21956/hrbopenres.15325.r42852

**Published:** 2024-11-13

**Authors:** Zulfiqarali G Abbas

**Affiliations:** 1 Muhimbili University of Health and Allied Sciences, Dar es Salaam, Tanzania

**Your Report**

 Title: Prevalence and incidence of peripheral neuropathy, peripheral artery disease, foot disease, and lower extremity amputation in people with diabetes in Ireland

 Strengths

1. Clear research aims and relevance: The protocol addresses a significant gap in diabetic foot disease epidemiology in Ireland, given the absence of a national diabetes registry. By providing prevalence and incidence estimates, this review could inform stakeholders about the need for targeted interventions.
2. Comprehensive Methodology: The protocol follows the PRISMA-P guidelines, with a structured approach to data collection, screening, and analysis.
3. Detailed inclusion and exclusion criteria: The well-defined criteria for population, settings, and study design ensure consistency and clarity in study selection, minimising bias.
4. Appropriate Data Extraction and Quality Assessment: The use of the Joanna Briggs Institute (JBI) tool for critical appraisal shows an attempt to ensure methodological rigor. The protocol also accounts for relevant factors like sociodemographic, urban versus rural location, and patient mobility.

Limitations and Areas for Improvement

1. Lack of Specific Quality Assessment Tools: While the protocol mentions the use of JBI tools, it doesn’t specify the exact tools for different study designs, which could limit clarity.
2. Limited Scope in Search Strategy: While the Coco Pop framework is used, the search strategy could be expanded to include more databases to ensure broader coverage. Limiting the search to PubMed, EMBASE, and Lenus might overlook grey literature that may not be present in mainstream databases.
3. Ambiguity in Meta-Analysis Plans: Although a meta-analysis is proposed if criteria are met, there is no indication of how data heterogeneity will be managed or the potential use of meta-regression to account for varying study characteristics.
4. Justification for Excluding Single-Centre Studies: Excluding single-centre studies may limit the review’s comprehensiveness. Including a rationale for this decision would strengthen the protocol.

Is the study design appropriate for the research question?

Yes

Is the rationale for, and objectives of, the study clearly described?

Yes

Are sufficient details of the methods provided to allow replication by others?

Yes

Are the datasets clearly presented in a useable and accessible format?

Yes

Reviewer Expertise:

Consultant Physician, Diabetologist and diabetic related foot complications

I confirm that I have read this submission and believe that I have an appropriate level of expertise to confirm that it is of an acceptable scientific standard, however I have significant reservations, as outlined above.

# Reviewer report: Reviewer response for version 2

**DOI:** 10.21956/hrbopenres.15325.r42034

**Published:** 2024-08-27

**Authors:** Zena Moore

**Affiliations:** 1 RCSI University of Medicine and Health Sciences, Dublin, Ireland

I have no new comments to make

Is the study design appropriate for the research question?

Yes

Is the rationale for, and objectives of, the study clearly described?

Yes

Are sufficient details of the methods provided to allow replication by others?

Yes

Are the datasets clearly presented in a useable and accessible format?

Yes

Reviewer Expertise:

Systematic review methods.

I confirm that I have read this submission and believe that I have an appropriate level of expertise to confirm that it is of an acceptable scientific standard.

# Reviewer report: Reviewer response for version 1

**DOI:** 10.21956/hrbopenres.15133.r39652

**Published:** 2024-06-03

**Authors:** Adrian H Heald

**Affiliations:** 1 Salford Royal Hospital, University of Manchester, Manchester, UK

Thank you for giving me the opportunity to review this protocol. I support the proposal.

**The authors are very modest about the implications for the findings of this excellent piece of work. Please can the abstract and overall conclusion reflect the international i,e global relevance of this piece of work**

**In relation to potential small improvements, re search terms it is important that 3 things be considered:**

  

1. Sociodemographic situation- [Ref-1,2]
2. Urban vs rural location- [Ref-3]
3. Mobility-[Ref-4]

The relevant papers (as above) to this that should be included in the Introduction

**Otherwise I am happy with the paper**

 Dr Adrian Heald

Is the study design appropriate for the research question?

Yes

Is the rationale for, and objectives of, the study clearly described?

Yes

Are sufficient details of the methods provided to allow replication by others?

Yes

Are the datasets clearly presented in a useable and accessible format?

Yes

Reviewer Expertise:

NA

I confirm that I have read this submission and believe that I have an appropriate level of expertise to confirm that it is of an acceptable scientific standard, however I have significant reservations, as outlined above.

## References

[1] : Foot ulceration and its association with mortality in diabetes mellitus: a meta-analysis. *Diabet Med* .2020;37(2) : 10.1111/dme.14151 211-218 10.1111/dme.14151 31613404

[2] : Social deprivation modifies the association between incident foot ulceration and mortality in type 1 and type 2 diabetes: a longitudinal study of a primary-care cohort. *Diabetologia* .2018;61(4) : 10.1007/s00125-017-4522-x 959-967 10.1007/s00125-017-4522-x 29264632 PMC6448990

[3] : Controlling antibiotic usage-A national analysis of General Practitioner/Family Doctor practices links overall antibiotic levels to demography, geography, comorbidity factors with local discretionary prescribing choices. *Int J Clin Pract* .2020;74(8) : 10.1111/ijcp.13515 e13515 10.1111/ijcp.13515 32306458

[4] : Sedentary behaviour is an independent predictor of diabetic foot ulcer development: An 8-year prospective study. *Diabetes Res Clin Pract* .2021;177: 10.1016/j.diabres.2021.108877 108877 10.1016/j.diabres.2021.108877 34058300

# Response

**Published:** 2024-08-16

**Authors:** Jennifer Pallin

**Affiliations:** University College Cork, Ireland

Dear Dr Heald,

 Thank you for taking the time for reviewing our protocol, and for your constructive feedback. As a result, revisions have been made, and points raised have been addressed below.

**Please can the abstract and overall conclusion reflect the international i,e global relevance of this piece of work**

 We have updated the abstract and introduction to emphasize the impact diabetic foot disease can have on individuals and health systems globally. Based on your recommendations we have included reference number 1 and 2 to support these points.

 However, we have not included reference 3 and 4, as we do not agree they strengthen the quality of the introduction section. The aim of reference 3 was to quantify the impact of different factors on levels of antibiotic prescribing choices by GP/family doctors. While we acknowledge this is an important area, particularly for people experiencing diabetic foot ulcers and infections, we do not believe inclusion of this paper strengthens the quality of the introduction section.  

 The aim of reference 4 was to prospectively explore the association between sedentary time (SED-time) and the development of diabetic foot ulcer (DFU) in people with diabetic peripheral neuropathy (DPN). While we acknowledge risk factors for diabetic foot ulcer onset is an important area for consider, we do not believe inclusion of this paper strengthens the quality of the introduction section. However, we acknowledge your point that we should emphasize the global relevance of this piece of work, and so we have included other internationally relevant pieces of work. We will also take this advice on board when writing the results paper.

**In relation to potential small improvements, re search terms it is important that 3 things be considered:**

  Sociodemographic situation- [Ref-1,2]

1. Urban vs rural location- [Ref-3]
2. Mobility-[Ref-4]

We thank the reviewer for the suggestion of additional factors to consider. As we have already completed the search using the terms indicated in version 1 of the protocol we have opted not to repeat the search with these as aspects as search terms. Instead, we have included additional points for data extraction, where information on these factors is available.

# Reviewer report: Reviewer response for version 1

**DOI:** 10.21956/hrbopenres.15133.r38450

**Published:** 2024-03-22

**Authors:** Zena Moore

**Affiliations:** 1 RCSI University of Medicine and Health Sciences, Dublin, Ireland

Many thanks for this protocol for a very important systematic review. The methods are in general clearly described, however, there are 2 minor issues for clarification. 1. In terms of data extraction,  given that you are interested in incidence as well as prevalence, length of patient follow up should be extracted, where this information is available. 2. In terms of quality appraisal, you are vague regarding which exact quality appraisal tool(s) will be  employed, I think you could provide some concrete examples, given that you have said that the following study designs are eligible for inclusion: Prospective and retrospective cross-sectional, case-control or cohort studies.

Is the study design appropriate for the research question?

Yes

Is the rationale for, and objectives of, the study clearly described?

Yes

Are sufficient details of the methods provided to allow replication by others?

Yes

Are the datasets clearly presented in a useable and accessible format?

Yes

Reviewer Expertise:

Systematic review methods.

I confirm that I have read this submission and believe that I have an appropriate level of expertise to confirm that it is of an acceptable scientific standard.

# Response

**Published:** 2024-08-16

**Authors:** Jennifer Pallin

**Affiliations:** University College Cork, Ireland

Dear Prof Moore,

 Thank you for taking the time for reviewing our protocol, and for your constructive feedback. Also, thank you for approving our protocol. However, based on your feedback, revisions have been made and points raised have been addressed below.

***In terms of data extraction,  given that you are interested in incidence as well as prevalence, length of patient follow up should be extracted, where this information is available***

 Based on your feedback, we have included length of follow-up in data extraction. We have ammended the protocol to reflect this.

***In terms of quality appraisal, you are vague regarding which exact quality appraisal tool(s) will be  employed, I think you could provide some concrete examples, given that you have said that the following study designs are eligible for inclusion: Prospective and retrospective cross-sectional, case-control or cohort studies.***

 Regarding quality appraisal, we acknowledge we were vague on exactly what JBI quality appraisal tool we would use. We have taken this feedback on board, and will make sure it is clearly stated in the final systematic review publication.
